# Accessibility of Treatment Among Women With Opioid Use Disorder: A Brief Review

**DOI:** 10.7759/cureus.27509

**Published:** 2022-07-31

**Authors:** Abdul Rahim Khan, Olubusola Olatunji, Danish Qureshi, Peterson Metellus, Stanley Nkemjika

**Affiliations:** 1 Psychiatry, Jinnah Medical and Dental College, Karachi, PAK; 2 College of Health Sciences & Human Services, Northern Kentucky University, Highland Heights, USA; 3 Psychiatry and Behavioral Sciences, Interfaith Medical Center, Brooklyn, USA; 4 Population Health Sciences, Georgia State University School of Public Health, Atlanta, USA

**Keywords:** health policy and advocacy, treatment disparity, womens health, addiction psychiatry, opioid use disorders

## Abstract

Opioid-use disorder (OUD) has become a social pandemic with a rising incidence and prevalence among women. Notably, women with OUD were more likely to have psychiatric comorbidities like major depressive disorder, anxiety disorder, and bipolar disorder. Evidence suggests that opioid exposure and subsequent disease among women compared to men is unique and attributable to hormonal estrogen levels. However, there remains a dearth of literature on their ability to access treatment when needed. There is also a gap in the perceived access to women as compared to men. Hence, our review will focus on factors that may affect women from seeking OUD treatment as compared to men.

## Introduction and background

Opioid-use disorder (OUD) has become a pandemic with a rising incidence and prevalence among women. According to the data collected by the National Survey on Drug Use and Health (NSUDH) in 2019, 10.1 million people, aged 12 or above, misused opioids in the past year [1]. Of these 10.1 million users, 9.7 million misused prescription pain relievers, and 745,000 used heroin [1]. Among those using prescription opioid pills, 9.3 million had only used prescription opioid pain relievers compared to 404,000 who had misused prescription pain relievers along with heroin in the past year [1]. This trend was supported by literature that noted a decline in prescription opioid use between 2015 and 2019 [2]. Furthermore, 2017 NSUDH statistics recorded that despite the burden of disease, about 10% of substance use disorder (SUD) clients or patients received any form of healthcare services.

Despite the increased prevalence of OUD in past decades, evidence in the literature reported that the male to female death rate attributable to OUD was 2.6% [3]. Similarly, the Center for Disease Control (CDC) also reported a 260% increase in drug-related overdose among women between 1999 and 2017 [4]. However, a variety of factors have been described in the literature to predispose women to develop this burden of disease and unwanted morbidity and mortality. Notably, women are reported to have an increased frequency of pain episodes, be diagnosed with pain-related disorders, and have a lower pain threshold [5]. One physiological factor that plays a role in response to opioid analgesics depends on the estrogen levels at the time. This is typified by studies that reported that changes in estrogen levels during the menstrual cycle are due to alterations in the availability of opioid receptors [6]. Another reason can further be supported by the variation and increased need for methadone in pregnant women receiving OUD treatment [7].

In the preliminary evidence report characterizing causes of severe withdrawals in opioid treatment, Dunn et al. reported women experienced more severe withdrawal symptoms than men [8]. Co-morbid mental health disorders also play a vital role in women with OUD, as evidence in the literature suggested that women with OUD, rather than those without OUD, were more likely to have psychiatric comorbidities like major depressive disorder, anxiety disorder, and bipolar disorder [9-10]. Similarly, considering that women are more prone to intimate partner violence (IPV), exposure to IPV has been shown to be associated with the likelihood of opioid misuse [11]. Another high-risk period is connected to the peripartal phase, which is a vulnerable period emotionally and physically among women [12]. Women have a higher rate of pharmacy-prescribed opioids as compared to men during the early postpartal phase, which further exacerbates the issue. Evidence in literature also showed that women of all ages were more likely than men to receive an opioid prescription refill. Notably, the reasons for these differences remain unknown. However, these findings could hypothetically be explained by the physical differences and physiological attributes of the pain thresholds that exist by biological sex. Other reasons include women are more likely to access healthcare, possess a higher prevalence of mental health disorders, or have a higher prevalence of certain chronic disease conditions for which opioids are commonly prescribed [13].

These multifactorial differences between men and women consequently mean unique challenges for treating OUD. In this review, we intend to discuss the most notable barriers in play that affect the accessibility of treatment among women with OUD. Hence, this brief report aims to highlight these barriers, address them, and suggest possible recommendations for making OUD treatment more accessible to women.

## Review

Loss to follow-up

Following a review of the literature, a few studies highlighted follow-up or referral-to-treatment rates after emergency department (ED) visits for opioid use. A qualitative study by Amaducci et al., which evaluated multiple factors affecting opioid use and treatment based on gender, reported that the warm hand-off referrals (direct referral) rate for women was 2.5% (2 of 81) as compared to men, which was 10.4% (11 of 106) [14]. They attributed this finding to a lack of childcare availability and family responsibilities. Another notable finding is the distance to the treatment facility, as it was reported as one of the major hurdles in accessing treatment, which leads to loss to follow-up in OUD patients [15]. It is noteworthy that a substantial percentage of those with a SUD visit to the ED were unhoused - 59% of them being women, with higher percentages of OUD than their non-homeless counterparts [16]. Hence, homelessness could be described as a major factor in losing OUD patients to follow-up [17].

Overdose prevention

Considering that overdose rates in women have increased tremendously in the past two decades [4], the role of biological sex differences has been implied in relation to naloxone treatment resistance [18]. In a retrospective study conducted in an emergency medical services set up between 2012 and 2014, which identified 124 opioid overdose patients, females were almost three times more likely not to receive naloxone than males (p-value 0.02) [18]. Females were also noted with more significant concern about using drugs associated with overdose and were perceived to be more receptive to overdose strategies [19]. They also showed higher retention of knowledge and technique from naloxone training compared to men [20]. The literature also noted that females expressed difficulty storing and carrying naloxone due to unstable housing [20-21]. Additionally, males were more likely to be referred to a pharmacological treatment program than females who were given psychotherapy referrals [22]. These suggest differences based on biological sex existent in managing and referring patients for overdose prevention.

Intimate partner violence

Women with a history of OUD and receiving treatment were more likely to be victims of IPV, as evidenced in the literature. A study that assessed 414 women undergoing methadone treatment for opioid withdrawal reported 47% being victims of physical or sexual IPV in the past six months, and 19% of these had suffered severe injuries from their intimate partners [23]. This factor was further elaborated in El Bassel et al.'s 2012 study on 3003 women enrolled in a methadone maintenance program (MMTP), which concluded that female IPV victims were more vulnerable to initiating or combining other illicit drugs [24]. Similarly, a study by Pallatino et al. reported IPV as a barrier to women undergoing OUD treatment successfully [25]. Furthermore, they reported that emotional abuse played a role in preventing females from being fully involved in their treatment recovery, as their partners used their history of OUD to lower their self-esteem [25]. Women also reported a profound negative impact of the lack of support from their partners in joining treatments. Finally, the role of spouse and peer support is undeniable as women quit treatment programs to join their using partners as well [26].

Motherhood

A ubiquitous factor that can present as a barrier for women seeking treatment for OUD is childcare and responsibility. Women were often the sole caretakers of the children, making it challenging to find the right kind of program. Studies have reported more than four out of every 10 adults with OUD are living with children [27]. Mothers and caregivers face stigma, a shortage of family-friendly treatment programs, and a lack of childcare [28]. The stigma of seeking OUD treatment among adults living with children is twice more likely to be a barrier than among adults without children [27]. This barrier effect increased to four times more likely when adjusted with demographic factors even though there is a clear understanding of perceived needs between caregivers with and without children [27]. Similarly, parents have described fear of being perceived as “bad” if their OUD is disclosed. This fear is not unfounded, as parents are also afraid of losing their children's rights after being labeled as unstable parents [26,29]. Additionally, mothers enrolled in treatment programs report that 50% of them had lost the custody of at least one child at some point, which led to severe hesitation in enrolling into programs and being labeled as 'unfit' parents [26]. An important role is played by media representation of parents with OUD, which leads to increased stigmatization [30]. Alternatively, OUD treatment facilities have a low provision (6%) in childcare [31], as adults with children were more likely to be attracted to OUD treatment programs with ease of access to transportation and availability of childcare [32-33].

Stigma to pharmacological treatment

Stigma exists concerning pharmacological treatment, which can prove to be a substantial barrier for many patients. This was described among staff at detoxification programs as unwilling to aid in starting young females on buprenorphine as reported in the literature. A similar response was seen among peer recovery support groups, where females were appraised or chastised for their sobriety while on buprenorphine/naltrexone, which led to discouragement and sudden attempt to stop opioid agonist treatment (OAT) [34]. This chastisement may lead to perceived stigma, especially for younger adults since they are more likely to be influenced by negative perceptions of trusted adults around them. However, the same stigma and thought have not been reported with naltrexone, which might be due to its antagonistic nature and no risk of diversion [34]. Another reason in play is the systemic preference for "abstinence-only" treatment with some cultural sentiments and moral nuance attached to substance use. Similarly, the stigmatization of SUD clients can be seen in practice among primary care physicians (PCP) and their non-verbal cues and attitudes towards their clients probably due to ‘counter-transference.’ This finding is supported by a national survey study that reported that less than 30% of PCP were willing to have a person with OUD marry into their family or be a neighbor, despite being enrolled in medication programs [35]. Another national survey in 2016 reported that the majority of PCP believed that SUD was a choice rather than a disorder, and people with OUD were more dangerous than the general population as they denied employment to those with OUD [36].

Pregnancy

Pregnant women with OUD are at high risk of adverse outcomes, including overdose and preterm birth [2]. Approximately 5.6 per 1000 live births are from mothers with opioid use during pregnancy [33]. It is crucial to address barriers to accessing quality care and treatment for pregnant females with OUD. Patrick et al. described a cross-sectional study where 67.6% secured a meeting in a buprenorphine clinic following an outpatient service appointment request for OUD treatment. Non-pregnant callers (73.9%) were more likely to be given an appointment as compared to pregnant females (61.4%) for buprenorphine-waived prescribers [33]. One of the possible explanations for limited program acceptance for pregnant females was that opinionated women's health clinicians had permission to prescribe buprenorphine, and less than 1% of obstetricians had received waivers to prescribe buprenorphine [37]. Also, 19.8% of clinicians on the list of waived clinicians provided on the Substance Abuse and Mental Health Services Administration (SAMHSA) website do not prescribe buprenorphine despite having waived status [33]. According to a 2016 study, only 12 states provided priority access to treatment programs for pregnant females, and only 19 states have specifically designed funded programs for pregnant women [38]. There is an urgent need to improve the availability of data and access to opioid treatment for all women, particularly pregnant females.

Financial difficulties

Feder et al. determined financial limitation as being one of the most reported factors in patients with a perceived unmet need for treatment (60.8%) [27]. According to a 2017 study, the most common source of payment for OUD treatment was the personal savings of patients [39]. However, this changed in most states after the 2014 Affordable Care Act, which allows the expansion of Medicaid as the primary form of insurance coverage seen in adults with OUD [39]. Even though Medicaid and other insurance options are now acceptable and available, there is a pattern where most clinicians decline insurance payments and ask for out-of-pocket cash, which is challenging for the unhoused population [33]. Similarly, female patients with private insurance are seen to be granted appointments more quickly than females with Medicaid for buprenorphine-waived prescribers (p< 0.001). The cost of initial outpatient appointments is $250 at buprenorphine-waived prescribers and $34 at opioid treatment programs (OTP) [33]. For patient groups comprising mainly the homeless population or those from low economic backgrounds, these costs can prove to be a massive hurdle in their quest for treatment.

Recommendations

Based on the review and commentaries discussed, we have summarized the most commonly faced barriers by women seeking help and treatment for OUD. Hence, we proscribe and enlist some recommendations that can address these issues: The availability of affordable treatment for everyone is the prime factor that will ease the approach to treatment. Women who are homeless or have childcare responsibilities have difficulty affording out-of-pocket treatments, private insurance, and/or accessing public insurance options like Medicaid. Hence, public insurance coverage for medication treatment for OUD should be easily accessible in all states and needs further expansion. Providing easy public access to evidence and treatment options will make a significant difference in the OUD burden by creating awareness and insight into the problem, hence making it easier for women to understand the available treatment options with the assistance of health care professionals. Continued education for all healthcare professionals, with contemporary evidence-based material regarding OUD treatment, will ensure a decrease in stigmatization among healthcare workers associated with OUD clients. This will further provide a more comfortable environment for women. Accessibility to OUD medication without labeling and stigmatization will make a significant difference, as this was typified following the COVID-19 pandemic relaxed methadone and buprenorphine regulations. For example, dispensing methadone out of pharmacies following the COVID-19 pandemic, instead of going to specific facilities, limited patient contact to the environment where they are labeled. This method has been suggested and implemented in developed economies. However, further proactive regulations to foster this trend are highly encouraged. Additionally, policies should consider the removal of stigma associated with women and mothers seeking treatment for OUD through education and awareness of the importance of treating women, especially mothers, to decrease poor outcomes in their children and improve maternal health outcomes. Finally, stringent policies on the privacy and anonymity of patients should also be formulated.

In contemporary times, social media is the prime medium of communication and promotion. Investing in the positive outcome-focused representation of OUD patients in video-graphic media will be helpful to sensitize the public. National education advertisement of OUD treatment facilities with a 24/7 online assistance chat could be immense toward catering to the malign. This educational incentive will provide a safe and private environment for patients to freely express their concerns before enrollment into treatment programs. This will stem the likelihood of negative judgment and stigmatization. Provision of an enabling environment for childcare assistance among mothers seeking treatment for OUD is highly advised. This will foster strong therapeutic alliances at both local and national levels by increasing family care at opioid treatment centers, hence providing a milieu environment for homeless or IPV mothers with the fear of losing child custody. Also, providing affordable transportation to the treatment facilities will remove the hurdle many women face, which can be achieved by giving discount public transportation for patients enrolled in specific facilities or through easy accessibility of medication at pharmacies. Inclusion of IPV questionnaire or screening among women seeking treatment should be encouraged. It should be identified and addressed in the screening phase and provide them with options and safe housing. Multi-disciplinary integrative approach programs are highly promoted and will assist in addressing the concerns of women in one visit. Finally, providers need continued re-orientation of the roles toward offering the most effective therapeutic approach despite their proscribed beliefs.

## Conclusions

Following our review, OUD remains one of the biggest challenges of chemical dependence in contemporary times. However, there remains a barrier concerning accessibility of treatment, which is more overt among females. Notably, females experience these barriers due to healthcare professionals' loss of follow-up mechanisms, poor financial sustainability, non-availability of supportive infrastructures like transportation, stigma rhetorics labeled on females with the disorder, the role of IPV on their self-esteem, and physical injuries. Based on the review, we have proffered some constructive recommendations that may lead to equitable access to OUD treatment of biological sex differences. These recommendations may assist with both community and national policy-based changes in treatment.
